# How to create an autism friendly hospital environment

**DOI:** 10.1192/j.eurpsy.2021.615

**Published:** 2021-08-13

**Authors:** F. Latif, S. King, A. Friedman, E. Valentine, K. Burley

**Affiliations:** 1 Psychiatry, Sidra Medicine, Doha, Qatar; 2 Psychology, Boston Children’s Hospital, Boston, United States of America

**Keywords:** autism, Child Psychiatry, Pediatric Hospital

## Abstract

**Introduction:**

Children with Autism Spectrum Disorder (ASD) struggle with communication, sensory sensitivities and social interaction. These difficulties can make hospital visits challenging. Every child with ASD is unique, and as such, some children can do well in clinical settings with minimal supports while others may require environmental modifications to achieve optimal care. ASD is prevalent worldwide and cultural differences can lead to varied care. Several hospitals, including Boston Medical Center in USA and Sidra Medicine and Research Center in Qatar, have attempted to address these challenges by developing strategies to create an ‘Autism Friendly’ environment.

**Objectives:**

This workshop will 1. Describe the 4 domains of an “Autism Friendly” environment 2. Describe practical steps for successful implementation of interventions and modifications to consider based on setting and culture.

**Methods:**

Didactic section 1 will describe the 4 domains for greating an ‘Autism Friendly environment’. Didactic section 2 will describe implementation in an inpatient and outpatient setting focusing on modifications based on environmental differences. These didactic presentations will be followed by a hands on, interactive section where participants will break out in small groups to learn specific implementation skills.

**Results:**

Participants will learn how to improve care offered to children with ASD during hospital visits. Participants will develop the skills to implement similar interventions in their home institutions.

**Conclusions:**

Hospitals can create an Autism Friendly environment by using 4 domains of intervention which could help improve provider skills and patient and family experience.

